# Green Nanodrugs: Research Progress and Challenges of Plant-Derived Nanovesicles in Tumor Treatment

**DOI:** 10.3390/pharmaceutics18030368

**Published:** 2026-03-16

**Authors:** Junsong Zhu, Xingyu Zhou, Qiong Lan, Jian He

**Affiliations:** 1 Guangxi Medical University, Nanning 530021, China; 2 State Key Laboratory of Targeting Oncology, Guangxi Medical University, Nanning 530021, China; 3 National Center for International Research of Bio-Targeting Theranostics, Guangxi Medical University, Nanning 530021, China; 4 Guangxi Key Laboratory of Bio-Targeting Theranostics, Guangxi Medical University, Nanning 530021, China; 5 Collaborative Innovation Center for Targeting Tumor Diagnosis and Therapy, Guangxi Medical University, Nanning 530021, China; 6 Guangxi Talent Highland of Major New Drugs Innovation and Development, Guangxi Medical University, Nanning 530021, China; 7 Targeting Theranostics Research Center of Guangxi Higher Education, Guangxi Medical University, Nanning 530021, China; 8 Pharmaceutical College, Guangxi Medical University, Nanning 530021, China

**Keywords:** plant-derived nanovesicles, cancer, nanomedicine, drug delivery

## Abstract

**Background**: Plant-derived nanovesicles (PDNVs), a class of naturally occurring nanoparticles with a phospholipid bilayer structure, have attracted significant attention in biomedicine, particularly in anti-tumor research, due to their broad source availability, low production cost, high biocompatibility, and low immunogenicity. **Methods**: This review systematically summarizes and analyzes the isolation methods, composition, anti-tumor mechanisms, and clinical translation potential of PDNVs based on literature retrieved from PubMed and Web of Science, with clinical trials identified and categorized using ClinicalTrials.gov. **Results**: Current research has made impressive progress in the application of PDNVs, both as direct therapeutic agents and as drug delivery systems. Their remarkable stability, ability to cross physiological barriers (e.g., the gastrointestinal tract and blood–brain barrier), and engineerability underpin their versatile potential. **Conclusions**: This review comprehensively outlines the compositional characteristics of PDNVs and explores their multi-dimensional mechanisms and application prospects as natural therapeutics and drug delivery platforms in cancer therapy. Despite challenges such as standardization in preparation, PDNVs represent a highly promising class of novel nanobiomaterials.

## 1. Introduction

Cancer is one of the deadliest diseases suffered by humans. According to the latest data from the Global Cancer Observatory (GLOBOCAN), as of 2022, there are about 20 million new cancer cases and 9.7 million cancer-related deaths each year [1], with one study estimating that, by 2050, new cancer cases will increase by an estimated 76.6% and cancer-related deaths will increase by 89.7% worldwide [2]. The American Cancer Society estimates that, in 2026, there will be an estimated 2,114,850 new cancer cases and 626,140 cancer deaths in the United States alone [3]. From 2005 to 2020, the total number of cancer-related deaths in China increased by 21.6%, to 2,397,772, and the number of years of life lost increased by 5.0%, to 56,598,975 [4]. On the other hand, from a socio-economic perspective, the comprehensive cost of cancer has posed a major challenge to the sustainable development of the country, as empirical data indicate that cancer imposes a substantial financial burden worldwide. Chinese patients incur costs ranging from $1840 to $14,572 per case; U.S. patients with private insurance face an average out-of-pocket expense of $592 per month after diagnosis; and European patients experience income loss in 56% of cases, along with additional medical or non-medical expenses in 86% of cases. These figures highlight the significant financial impact of cancer across different health systems [5,6,7]. From 2020 to 2050, the total global economic cost of cancer is estimated to reach US$25.2 trillion, equivalent to approximately 0.55% of the annual tax revenue of each country [8]. Therefore, in the context of the continuous increases in cancer incidence and mortality, controlling medical costs while improving treatment effectiveness has become a core issue that must be solved urgently in the global public health system.

Although surgical resection, radiotherapy, chemotherapy, targeted therapy, and immunotherapy have formed the core system of contemporary oncological treatment and have made significant progress in improving patient outcomes, clinical practice still faces many significant challenges [9,10,11]. Traditional therapies, such as radiotherapy and chemotherapy, often work on rapidly proliferating tumor cells but are not very effective in distinguishing between cancerous and normal cells, leading to a wide range of systemic side effects such as bone marrow suppression, gastrointestinal mucositis, and long-term fatigue, which seriously affect treatment tolerance and quality of life [12,13]. To make matters worse, the inherent heterogeneity and genetic instability of tumor cells often lead to the development of acquired resistance, which ultimately leads to the failure of initially effective chemotherapy or targeted therapy [14,15]. Although immunotherapies such as immune checkpoint inhibitors have brought hope for long-term survival for some patients with advanced tumors, their overall response rate still needs to be improved, and may cause a series of unique immune-related adverse events from mild rash to severe immune-related pneumonia and myocarditis, with greater uncertainty in time, duration and severity, and more complex management [16,17]. In addition, factors such as the imbalance of medical resources and diagnosis and treatment levels in different regions, as well as the lack of popularization of genetic testing, also limit the full implementation of the concept of precision medicine in clinical practice, making it difficult for some patients to obtain the most suitable individualized treatment plan. Therefore, today’s cancer treatment urgently needs more economical, safer, and less adverse treatment options. The above problems combine to show that it is difficult to fundamentally break through the existing treatment ceiling by relying only on the improvement of drug molecules themselves, and there is an urgent need for a new technology path that can intervene at the level of delivery and bioregulation at the same time.

In this context, nanotechnology has been gradually introduced into the field of cancer treatment research, and its core advantage is that it can improve drug stability, target enrichment, and responsive regulation of the tumor microenvironment through scale regulation, so as to make up for the systemic shortcomings of traditional treatment strategies to a certain extent. Among various nanotechnologies, bio-derived nanovesicles have attracted increasing attention due to their intrinsic biocompatibility and ability to deliver functional biomolecules directly to target cells. In particular, plant-derived nanovesicles (PDNVs), as an emerging class of bio-nanomaterials, have experienced leapfrog development from basic exploration to application transformation in the field of tumor treatment, and have gradually become an important strategy to subvert the traditional treatment paradigm [18,19]. Compared with synthetic nanocarriers, the core competitiveness of PDNVs stems from their “natural-intelligent” dual recombination characteristics. From a lipidomics perspective, the lipid bilayer membrane of PDNVs is rich in glycerides, sphingolipids, and glycerophospholipids, which give them excellent membrane stability and cross-species biocompatibility, and some unique phospholipid components can also confer additional biological functions [20]. In fact, PDNVs were discovered earlier than animal exosomes. In the 1960s, scientists had already discovered vesicle-like structures in plant cells through electron microscopy [21]. In recent years, more and more studies have reported the extraction of plant nanovesicles from plants such as *Grapefruit* [22], *Rice bran* [23], and *Pueraria lobata* [24], and plant vesicles of different plant origins exert beneficial therapeutic effects in different diseases. Several recent reviews have summarized the biological characteristics and potential therapeutic applications of PDNVs, primarily in non-oncological settings [25]. These studies mainly discuss their possible roles in inflammatory, intestinal, and metabolic disorders, where PDNVs have been reported to participate in immune modulation, gut microbiota regulation, and oxidative stress alleviation [26]. Their natural origin and biocompatibility have also been considered advantageous for drug delivery applications [27].

Small molecule metabolomics analysis showed that PDNVs can serve as natural reservoirs for phytochemicals, including polyphenols, terpenes, and alkaloids, and synergistically regulate multiple pathways such as PI3K-Akt to play a tumor suppressive role [28,29,30]. Nucleic acid metabolomics analysis revealed that PDNVs can directly regulate gene expression in tumor cells or macrophages through the delivery of plant-derived microRNAs, mtDNA, etc [31,32,33]. On the other hand, from a production perspective, PDNVs also have the characteristics of a wide range of raw materials, low production costs, and can be mass-produced, and have great potential and broad prospects in the field of cancer applications. Therefore, this review reviews the research progress related to PDNVs, and its continuous development in tumor treatment is of great significance. However, despite the rapid expansion of PDNV-related research, existing reviews have primarily emphasized their general biological characteristics or applications in non-cancer diseases, whereas dedicated and cancer-focused reviews are relatively limited [34,35]. In particular, the anticancer mechanisms of PDNVs have not yet been comprehensively categorized or systematically summarized. Therefore, this review focuses on recent advances in PDNV research within oncology, aiming to provide a systematic categorization of their anticancer mechanisms and to clarify current research progress in this rapidly evolving field.

## 2. Germination of PDNVs

**Figure 1:**
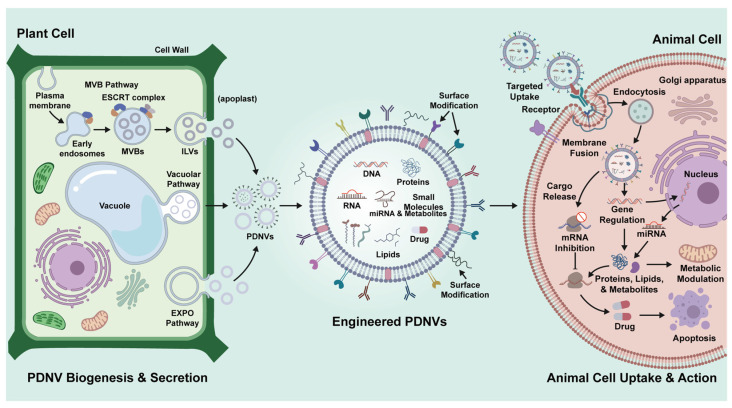
Biogenesis and cross-kingdom regulation of plant nanovesicles. Plant nanovesicles are secreted from plant cells via the MVB pathway, vacuolar pathway, or EXPO pathway, and with or without artificial modification, they deliver lipids, proteins, nucleic acids, small molecule metabolites, or drugs across kingdoms to animal organisms.

Compared with animal cells, plant cells have significant differences in the biogenesis pathway of nanovesicles due to their unique subcellular structure (such as central large vacuoles, cell walls, etc.). Although the current research on the biogenesis pathway of plant nanovesicles is relatively limited, several models have been proposed to elucidate their secretion mechanisms, including the multivesicle (MVB) pathway, the vacuole pathway, and the exocyst-positive organelle (EXPO) pathway (Figure 1) [36]. The MVB pathway is a vesicle secretion pathway common to mammals and plants, and usually contains two stages: Firstly, the cytoplasmic membrane buds inward to form early endosomes. Subsequently, the transport essential endosome sorting complex (ESCRT) sorts the membrane cargo to the invagination of the membrane, and finally forms intraluminal vesicles (ILVs) in the MVB. The MVB then fuses with the plasma membrane to secrete the vesicles outside the cell [37,38,39]. The vacuolar pathway is an immune response of plant cells to fight foreign infections. It has been reported that when pathogenic bacteria invade, *Arabidopsis* cells can release antimicrobial proteins and phenolic compounds outside the cell through the fusion of the vacuolar membrane with the plasma membrane to resist external infection [40]. Accumulating experimental evidence demonstrates that the exocyst-positive organelle (EXPO) is a plant-specific double-membrane compartment marked by Exo70E2, which sequesters cytosolic cargos and undergoes direct fusion of its outer membrane with the plasma membrane to release internal single-membrane vesicles into the extracellular space, thereby defining an unconventional secretion pathway that operates independently of the multivesicular body (MVB) route and the canonical ER–Golgi secretory system and contributes to the extracellular delivery of immune- and stress-associated molecules [41,42]. From a broader perspective, available findings suggest that the biogenetic landscape of plant nanovesicles remains far from fully resolved, and future studies employing higher-resolution analytical strategies and pathway-specific markers will be required to disentangle nanovesicles originating from distinct secretion routes and to clarify whether such biogenetic diversity translates into functional divergence.

## 3. Isolation and Purification of PDNVs

**Figure 2:**
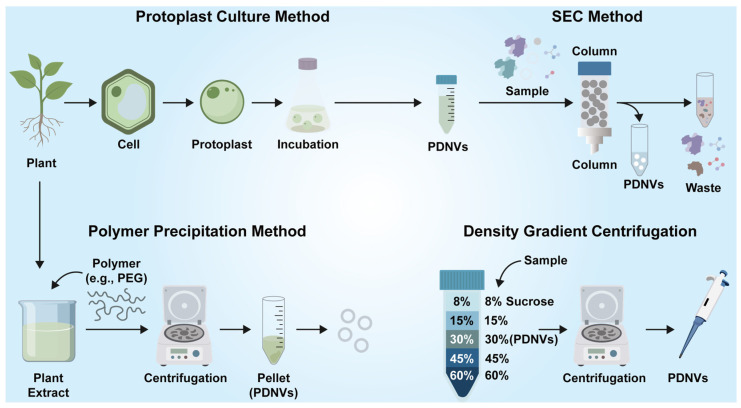
Extraction methods of plant nanovesicles.

The isolation and purification of plant nanovesicles (PDNVs) is a key prerequisite for elucidating their biological functions and application potential, and their technical strategies need to overcome the special complexity of plant samples. Compared with mammalian exosomes, they cannot be directly isolated from serum, body fluids, cell culture supernatants and other body fluids [43,44,45]. Factors such as complex plant cell wall structure, differences in vesicle physicochemical properties, and high polysaccharide and secondary metabolite interference require the development of specific optimization strategies (Figure 2). At present, the separation of PDNVs mainly relies on physical centrifugation, size exclusion chromatography, polymer precipitation and ultrafiltration, among which differential centrifugation combined with density gradient purification is still the core scheme, but the specific operation needs to be adapted to the characteristics of plant samples.

### 3.1. “Gold Standard” Ultracentrifugation

Ultracentrifugation is usually used to wash the plant tissue and then crush and squeeze the juice; large particles of impurities are removed by low-speed centrifugation (500–2500× *g*); organelle fragments, fibers and proteins are removed by medium-speed centrifugation (10,000–20,000× *g*); and finally ultracentrifugation (100,000–200,000× *g*) is used to precipitate the crude extract of vesicles [46]. Therefore, density gradient centrifugation can be further applied for purification—the sucrose gradient (8–60%) remains the classical approach, and according to most reports, PDNVs are typically enriched in the 30–45% sucrose layer, corresponding to a density of approximately 1.13–1.21 g/mL, although the exact range may vary depending on plant species and preparation conditions [47,48]. In recent years, the method of obtaining *Catharanthus roseus* (L.) Don-derived nanovesicles based on protoplast culture technology have been further optimized, and their core processes include cellulase and pectinase, which are used to synergistically dissociate the cell wall and release biologically active protoplasts in the enzymatic hydrolysis pretreatment stage. The protoplasts were then suspended in optimized medium and cultured for 7 days under continuous dark conditions to mimic the natural biogenesis of vesicles in plant cells and promote extracellular secretion. Finally, the nanovesicles in the supernatant were enriched by differential centrifugation combined with ultracentrifugation [49]. Therefore, despite its inherent limitations—such as the potential for high centrifugal forces to induce vesicle aggregation, deformation, or partial loss of biological activity, and the co-precipitation of non-vesicular components like protein complexes and ribonucleoproteins with PDNVs—ultracentrifugation remains an indispensable benchmark, due to its procedural stability, well-established workflow, and extensive historical application, against which the performance of alternative PDNV isolation techniques is evaluated.

### 3.2. Other Separation Methods

Size exclusion chromatography (SEC), as a representative technique for native separation, can achieve gentle sorting based on hydrodynamic diameter (PDNVs are mostly distributed at 30–200 nm), which can better retain the biological activity of vesicles [22,50]. In addition, polymer-based precipitation methods, such as polyethylene glycol (PEG), are frequently applied owing to their operational simplicity and relatively high recovery efficiency [51,52]. However, because plant nanovesicles are typically isolated from mechanically disrupted plant tissues, the resulting extraction solutions are highly complex, containing abundant proteins, polysaccharides, and other soluble biomolecules. As a consequence, individual isolation techniques often fail to achieve sufficient purity, and combinatorial purification strategies are therefore commonly adopted to maximize nanovesicle enrichment [53,54]. To provide a comprehensive overview, Table 1 summarizes the main isolation strategies for plant-derived nanovesicles, highlighting their respective advantages and limitations. This comparison underscores that while single techniques offer specific benefits, combinatorial approaches are often necessary to balance yield, purity, and vesicle integrity. Nevertheless, despite the increasing availability of alternative separation approaches, ultracentrifugation-based protocols remain the most important reference standard in the field. Owing to their methodological maturity, broad applicability, and high reproducibility across different plant species and experimental systems, traditional methods such as ultracentrifugation continue to provide the core comparative framework for evaluating newly developed plant nanovesicle isolation technologies.

## 4. Characterization of PDNVs

### 4.1. Physical Properties

The physical characterization of PDNVs is the basis for their functional research and clinical application, and transmission electron microscopy (TEM) and scanning electron microscopy (SEM) can be used to verify the morphology and size of PDNVs [55,56]. Dynamic light scattering (DLS) can be used to measure the hydrodynamic diameter of vesicles (typical range of 50–200 nm) and dispersion index (PDI < 0.3 suggests monodispersity) as a rapid screening method, but the residual polysaccharides/polyphenols in plant samples are prone to light scattering interference, so it is necessary to use ζ potential analysis (usually −15 to −30 mV) to support the surface charge stability [57]. Nanoparticle tracking analysis (NTA) inverts particle size distributions using Brownian trajectories in that single-particle resolution distinguishes PDNVs (peak 1: 80–150 nm) from co-separated protein aggregates (peak 2: <50 nm), and fluorescent labeling patterns (e.g., membrane dye DiR) specifically exclude non-vesicular background signals [58].

### 4.2. Lipids

As an important component of plant cells, these lipids not only play a key role in providing structural integrity and acting as metabolic energy reserves, but also, because of their unique compositional characteristics, PDNVs show multifaceted value as a class of extracellular vesicle-like nanostructures derived from plant cells [31,59]. Their signature lipid bilayers not only constitute a basic physical barrier, but also endow them with excellent biocompatibility, low immunogenicity, and intrinsic targeting and drug delivery capabilities through their unique lipid composition, which together lay a broad application prospect for PDNVs in biomedical fields such as tumor therapy. Lipid components not only play a structural supporting role, but also actively participate in the regulation of membrane stability, microenvironment response, cell recognition and internalization processes, and the construction of drug delivery systems [60]. First, the lipid bilayer structure of plant nanovesicles exhibits a high degree of compatibility with mammalian cell membranes, which allows them to efficiently deliver the active substances they carry (such as citrate) to tumor cells through the macropinocytosis pathway, thereby overcoming the biological barrier challenges faced by many traditional synthetic nanocarriers [61]. Secondly, the unique lipid components in PDNVs, such as 6-gingerol, 8-gingerol, 10-gingerol, 6-shogaol, and 8-shogaol, make PDNVs have significant tumor cell inhibition and tumor growth inhibition effects [20]. In a research model for liver cancer, an engineered plant nanovesicle platform loaded with sorafenib has been shown to encapsulate and reduce the leakage of sorafenib in the gastrointestinal environment and enhance its ability to cross the intestinal epithelium for effective liver accumulation [62]. Of particular note, hybrid nanovesicles constructed through cross-border membrane fusion strategies (such as fusing cancer cell membranes and plant vesicle membranes) further endow these vectors with homologous targeting capabilities, enabling them to accurately identify and enrich in tumor tissues and achieve multi-level regulation of the tumor microenvironment [63].

### 4.3. Protein

The proteins encapsulated in different PDNVs have unique characteristics due to different species, vesicle particle sizes and preparation methods, covering enzymes, signal peptides, transmembrane proteins and a variety of bioactive factors, which exert synergistic anti-cancer effects by directly acting on tumor cells or indirectly regulating the tumor microenvironment. Proteomic profiling of nanovesicles derived from edible *tea* flowers identified a diverse repertoire of proteins, including oxidative stress-associated enzymes, metabolic regulators, and membrane-related proteins. Notably, a subset of these proteins shows homology to conserved eukaryotic protein families and shares structural or functional similarity with human proteins involved in redox regulation and cellular homeostasis. These findings suggest that *tea* flower-derived nanovesicles encapsulate evolutionarily conserved protein cargos with potential cross-kingdom biological relevance [28]. Extracellular nanovesicles (SMDENVs) were successfully isolated from *Shiitake mushroom*, and 1290 proteins were identified, among which the significant enrichment of key functional proteins such as transmembrane proteins, heat shock proteins 90/70 and phospholipase C not only endowed SMDENVs with excellent stability over conventional liposomes, but also directly mediated their effective internalization by Caco-2 cells [64]. This provides a solid theoretical basis for the development of PDNVs as natural nanodrugs or functional food components [65]. In addition, some researchers believe that three plant proteins, heat shock protein 70 (HSP70), S-adenosine-homocysteinase and glyceraldehyde 3 phosphate dehydrogenase (GAPDH), have been detected in exosomic analysis of a variety of plant vesicles and even animals, suggesting that nanovesicles of different origins have some common characteristics [66]. Although the study of plant nanovesicles has been conducted for many years, there is still a lack of characteristic proteins as identification indicators for plant nanovesicles.

### 4.4. Nucleic Acid

As an emerging natural nanocarrier, the anti-tumor efficacy of PDNVs is not only due to active components such as proteins and lipids, but also due to the rich and diverse nucleic acid components they carry. PDNVs encapsulate a variety of nucleic acid substances, including microRNA (miRNA) and plant mitochondrial DNA (mtDNA), which are effectively protected by the phospholipid bilayer structure of PDNVs to avoid degradation by enzymes, and then efficiently delivered to mammalian tumor cells or immune cells in the tumor microenvironment, playing an important regulatory role. High-throughput sequencing of *Brucea javanica* vesicles revealed multiple endogenous miRNAs, including ten conserved species with sequence similarities to mammalian miRNAs, highlighting their potential for cross-kingdom interactions [67]. *Glycyrrhiza uralensis* Fisch roots-derived vesicles naturally encapsulate conserved miRNAs such as miR-2916, which are stably incorporated and represent a conserved class of plant small non-coding RNAs [68]. *Broccoli*-derived extracellular vesicles carry miR-167a, whose abundance can be enhanced through selenium biofortification, emphasizing the plasticity of nucleic acid content in response to environmental or nutritional factors [69]. In addition to small RNAs, *Artemisia annua*-derived vesicles contain mitochondrial DNA, illustrating that PDNVs can deliver a broader spectrum of nucleic acids beyond miRNAs [33]. Although plant nanovesicle-mediated nucleic acid delivery represents a highly promising field, our understanding of gene expression patterns and metabolic regulatory networks across the vast plant kingdom remains limited and fragmented. Plant gene expression and metabolite accumulation are strongly influenced by geographical and environmental conditions. Consequently, whether nanovesicles derived from plants of different regions, altitudes, and cultivated or wild origins share consistent nucleic acid composition and enrichment levels remains an important open question worthy of in-depth investigation.

### 4.5. Specific Metabolites

In addition to the above components, another important class of PDNVs is the specific metabolites encapsulated in them, such as flavonoids, terpenoids, organic acids, and sterol derivatives. These metabolites also exhibit multi-target anti-tumor efficacy, and their mechanism of action is closely related to metabolomics characteristics. The phospholipid bilayer membrane structure of PDNVs can stably encapsulate these lipophilic metabolites and promote their efficient uptake by tumor cells, thereby experimenting with cross-border regulation. For example, oral exosome-like nanovesicles from *Phellinus linteus* carry pharmacologically active small molecules such as triterpenoids and polysaccharides, which have been associated with anti-cancer effects [70]. *Cucumber*-derived vesicles contain metabolites including cucurbitacin B, emphasizing their role as natural carriers of bioactive compounds in cancer-related applications [71]. Non-targeted metabolomics analysis revealed that the excellent activity of nanovesicles of different sizes against the proliferation and migration of oral squamous cell carcinoma was closely related to the significant enrichment of specific anti-tumor-related small molecule metabolites in vesicles [72]. Exosome-like nanovesicles from *Hypericum perforatum* serve as a promising natural photosensitizer with notable anti-tumor potential and good biocompatibility [73]. These studies collectively show that plant-derived nanovesicles (PDNVs) can not only effectively encapsulate, stabilize, and deliver endogenous specific plant metabolites through their unique phospholipid bilayer structure, but also synergistically utilize the multi-target pharmacological mechanisms of these active ingredients to achieve efficient and biocompatible anti-tumor treatment, highlighting their great potential as a new generation of intelligent natural nanodrug carriers.

## 5. Antitumor Mechanism of PDNVs

### 5.1. Direct Killing Effect of Tumors

PDNVs have shown significant direct anti-tumor activity in the field of tumor therapy, and their mechanisms of action involve inducing apoptosis, blocking the cell cycle, disrupting redox homeostasis, and inhibiting energy metabolism. Multiple studies have confirmed through in vitro and in vivo models that PDNVs of different plant sources can directly act on tumor cells through the specific bioactive components they carry, inhibiting their proliferation and survival. Exosome-like nanovesicles (ACNVs) derived from *Asparagus cochinchinensis* can inhibit tumor cell proliferation by directly inducing apoptosis in hepatocellular carcinoma cells, and their mechanism of action is closely related to the activation of apoptosis-related factors AIF, Bax, and Bak, which in turn trigger caspase-9 and cleave key cellular proteins such as PARP, and this process has low toxicity to normal hepatocytes, showing good tumor selective inhibition properties [74]. Similarly, exosome-like nanovesicles derived from *Citrus limon* can selectively activate the exogenous apoptotic pathway by specifically upregulating the expression of TRAIL (tumor necrosis factor-associated apoptosis-inducing ligand) and its death receptor DR5 in tumor cells, thereby directly inhibiting the proliferation of multiple tumor cells and effectively inhibiting tumor growth in chronic myeloid leukemia xenograft tumor models [75]. In addition, *onion*-derived nanovesicles (ODNVs) selectively inhibit the proliferation of prostate cancer (PC-3) and cervical cancer (HeLa) cells by inducing the mitochondrial apoptosis pathway and inducing S-phase cell cycle arrest, highlighting their mechanism of exerting anti-cancer effects by directly inducing apoptosis in tumor cells [76]. Through integrated metabolomics, network pharmacology and experimental validation, it was systematically elucidated for the first time that exosome-like nanoparticles (GELNVs) derived from *Garcinia mangostana* L. synergistically regulate key targets of PI3K-Akt/MAPK signaling pathway (such as AKT1 and MAPK1) through their rich flavonoids, alkaloids and other multi-active components, thereby inducing glioma cell apoptosis, inhibiting proliferation and promoting the polarization of microglia to anti-tumor M1 phenotype [29]. Exosome-like nanoparticles rich in trans-δ-glucosin-derivatives isolated from *grape* cell cultures can act as natural bioactive carriers to produce dose-dependent selective killing of triple-negative breast cancer cells MDA-MB-231 by inducing G1 phase cell cycle arrest and apoptosis, while the toxicity to normal cells is weak [30]. Exosomal nanovesicles (ADNVs) derived from *Centella asiatica* can be internalized by HepG2 cells, selectively inhibiting the proliferation and promoting apoptosis of hepatocellular carcinoma cells by inducing reactive oxygen species (ROS) accumulation, causing mitochondrial damage, inducing G1 cell cycle arrest, and regulating key metabolic pathways such as amino acid metabolism and lipid biosynthesis [32]. Despite these encouraging observations, evidence supporting the direct antitumor effects of PDNVs remains predominantly preclinical and methodologically diverse. Differences in plant species, vesicle isolation protocols, and compositional definition substantially impair reproducibility and limit cross-study comparability, while the extent to which observed effects arise from vesicle-associated cargos versus co-extracted phytochemicals remains difficult to resolve. Furthermore, most mechanistic insights are derived from in vitro assays or xenograft models with limited predictive value for human tumors, underscoring the need for standardized preparation strategies and validation in more clinically relevant models before firm therapeutic conclusions can be drawn.

### 5.2. Immunomodulatory Effects

In addition to direct cytotoxic effects, some plant components can also indirectly inhibit tumors by regulating gene expression and the immune microenvironment. *Ginseng*-derived nanoparticles (GDNPs) can activate the TLR4/MyD88 signaling pathway on the surface of macrophages through their ceramides, promoting the polarization of tumor-associated macrophages to the M1 phenotype, and the polarized M1 macrophages produce reactive oxygen species to kill tumor cells and further recruit immune cells such as CD8+ T cells to infiltrate, thereby inhibiting the growth of melanoma [77]. *Platycodon grandiflorum*-derived extracellular vesicles can directly trigger apoptosis by inducing a large amount of reactive oxygen species in tumor cells and reverse the immunosuppressive tumor microenvironment, while regulating the intestinal flora to enhance systemic anti-tumor immunity, thereby synergistically inhibiting the growth of triple-negative breast cancer in multiple dimensions, indicating that plant nanovesicles are effective both orally and intravenously [78]. *Garlic*-derived nanoparticles specifically activate intestinal γδ T cells, enhance their production of interferon-γ (IFN-γ), and promote their trafficking to tumor sites via the CXCR3-CXCL10 axis, thereby remodeling the tumor immune microenvironment and eliciting robust synergistic antitumor effects in combination with anti-PD-L1 immune checkpoint blockade. Notably, these effects are more pronounced with vesicle-encapsulated formulations than with lysates or crude extracts, highlighting the functional advantage conferred by the nanoparticulate vesicular structure in improving bioavailability and immunomodulatory efficacy [79]. Plant nanovesicles (I-PDNVs) derived from indole-3-butyric acid (IBA)-induced regeneration of *Cannabis sativa* roots activate dendritic cells to promote their maturation and enhance antigen presentation through the TLR2/TLR4 signaling pathway, and then synergistically activate adaptive immune responses such as natural killer (NK) cells, Th1 cells, and cytotoxic T lymphocytes (CTLs). At the same time, as a vaccine vector, it can effectively induce potent antigen-specific CTL responses, reduce the proportion of regulatory T cells (Tregs) and CD8^+^ T cell depletion in the tumor microenvironment, thereby exerting a multidimensional antitumor effect [80]. *Artemisia annua*-derived nanovesicles (ADNVs) can serve as an efficient cross-border delivery vector to deliver the mitochondrial DNA (mtDNA) carried by them to tumor-associated macrophages (TAMs), and drive macrophages to polarize from the pro-tumor M2 phenotype to the anti-tumor M1 phenotype by activating the cGAS-STING intrinsic immune signaling pathway, thereby reshaping the tumor immune microenvironment and inhibiting tumor growth. And it shows a significant synergistic effect with PD-L1 blockade therapy [33]. Exosome-like nanovesicles (YQ-ELNVs) were isolated from the multi-herb traditional Chinese medicine formula Yiqi Huoxue Jiedu decoction—comprising herbs such as *Astragalus membranaceus*, *Salvia miltiorrhiza*, and *Hedyotis diffusa*—which is used as a therapeutic approach for ovarian cancer. These YQ-ELNVs were shown to synergistically regulate the polarization balance of M1/M2 macrophages and optimize the ratio of CD4^+^/CD8^+^ T cells by delivering functional miRNAs (e.g., miR-8783) and bioactive lipid constituents. Through these mechanisms, YQ-ELNVs effectively reshape the immunosuppressive tumor microenvironment and inhibit ovarian cancer growth [81]. Collectively, these studies suggest that plant-derived nanovesicles exert antitumor effects through coordinated modulation of both innate and adaptive immunity. However, in many cases, immune mechanisms are primarily inferred from phenotypic markers or cytokine alterations, and the direct causal contribution of specific immune subsets to tumor control requires further clarification. In addition, the durability, specificity, and potential regulatory feedback of vesicle-induced immune activation remain incompletely characterized. Future studies incorporating more rigorous immune-depletion strategies, longitudinal analyses, and pathway-specific validation would help strengthen the mechanistic understanding and translational potential of these immunomodulatory effects.

### 5.3. Synergistic Regulation via Metabolic Modulation

PDNVs also show significant potential in alleviating toxic side effects caused by chemotherapy and radiotherapy, and their mechanisms of action involve antioxidant, anti-inflammatory, organ-specific protection, and fine regulation of cell death pathways. Firstly, in the field of radiation protection, exosome-like nanovesicles derived from *Biyang floral mushroom* (BFMELNs) showed excellent multi-pathway protective efficacy. Studies have shown that BFMELNs not only have good temperature tolerance and gastrointestinal stability, but also can be effectively taken up by intestinal epithelial cells and hepatocytes through clathrin and dynein-mediated endocytic pathways, which in turn scavenge free radicals through the polyphenols and flavonoids they carry, and upregulate the activities of superoxide dismutase and glutathione peroxidase, significantly reducing oxidative stress and DNA damage caused by ionizing radiation, and effectively restoring peripheral blood counts and protecting liver, spleen and other organs in animal models [57]. Secondly, for specific organ damage caused by chemotherapy drugs, nanovesicles derived from different plants showed precise intervention effects: in the cisplatin-induced model of acute kidney injury, *Honeysuckle*-derived nanovesicles can effectively accumulate in the kidney, by reducing the levels of pro-inflammatory factors IL-1β, IL-6 and TNF-α, while upregulating the anti-inflammatory factor IL-10, and protecting the mitochondrial function of renal tubular epithelial cells, thereby significantly improving serum creatinine and urea nitrogen levels, and highlighting its nephroprotective effects mediated via a dual “anti-inflammatory and mitochondrial protection” pathway [82]. Importantly, the mitigating effect of plant nanovesicles on cardiotoxicity reveals a deeper molecular pathway: *Momordica charantia* L.-derived nanovesicles provide a new strategy to combat doxorubicin-based cardiotoxicity, the core mechanism is to stabilize the expression of autophagy adapter protein p62 and inhibit its ubiquitination degradation, thereby activating the Keap1-NRF2 antioxidant signaling axis, enhancing the antioxidant capacity of cardiomyocytes, and reducing oxidative stress and apoptosis caused by doxorubicin [83]. Coincidentally, another study also confirmed that *Momordica charantia*-derived nanovesicles can significantly reduce radiation-induced cardiomyocyte damage in vitro and in vivo models by scavenging excess reactive oxygen species, mitigating DNA damage and mitochondrial dysfunction, and activating the AKT/ERK signaling pathway [84]. Taken together, these studies jointly confirm the great potential of plant nanovesicles as adjuvant therapeutic agents, which precisely intervene on the molecular mechanisms of different toxic side effects of radiotherapy and chemotherapy through their unique active ingredients and signaling pathways, opening up new directions for the development of efficient and low-toxicity tumor treatment adjuvant strategies. Although numerous studies have demonstrated the potential of plant-derived nanovesicles (PDNVs) to be incorporated into cancer therapy in various forms as an adjuvant, it is important to note that most evidence is still derived from short-term animal studies. Given that cancer is a chronic disease, clinical treatment often extends over long periods, and long-term maintenance and tumor microenvironment remodeling may be required even after the primary treatment phase. Therefore, whether PDNVs possess the long-term safety and stability necessary to serve as a consistent adjuvant or supplement throughout extended therapeutic regimens remains a critical and unresolved question.

To systematically summarize their modes of action, we have categorized the antitumor mechanisms of plant-derived nanovesicles (PDNVs) into three preliminary classes—apoptosis regulation, immunomodulation, and metabolic intervention—as integrated in Table 2. As summarized, PDNVs exert antitumor effects primarily through direct cytotoxicity (e.g., induction of apoptosis, etc.); immune modulation (e.g., regulating various immune cell functions and responses, etc.); and synergistic protective effects (e.g., antioxidant activity, etc.), which collectively enhance therapeutic efficacy. Together, these findings highlight the multifunctional potential of PDNVs as anticancer agents.

## 6. As a Drug Delivery System

With their unique natural advantages, PDNVs have become highly competitive candidates for next-generation drug delivery vectors. Its typical phospholipid bilayer structure is not only a natural drug encapsulation carrier, but also shows significant advantages over traditional synthetic nanocarriers (e.g., liposomes, polymer micelles) and mammal-derived exosomes in multiple dimensions. Specifically, PDNV has the characteristics of a wide range, low cost and relatively simple preparation, while completely avoiding the risk of human exosome-borne diseases and has higher safety [85]. Their natural lipid composition and surface properties give them excellent structural stability, which can effectively protect the loaded drugs (including small molecule drugs and nucleic acids) from enzymatic degradation under different pH and temperature conditions, thereby significantly improving the bioavailability and delivery efficiency of drugs.

Currently, exogenous therapeutic molecules can be efficiently loaded onto the cavities or membrane structures of PDNVs through a variety of physical (e.g., sonication, extrusion), chemical, and biological strategies (e.g., membrane fusion techniques), further expanding their application potential. In terms of specific applications, the researchers successfully constructed a biomimetic drug delivery system by attaching heparin-based nanoparticles (DNs) loaded with doxorubicin and modified with cRGD-targeted peptides to the surface of natural *Grapefruit* extracellular vesicles. The system can effectively cross the blood–brain barrier/blood–tumor barrier and achieve efficient enrichment in glioma tissues with the help of cRGD’s active targeting, ultimately demonstrating excellent anti-tumor efficacy [86].

In another study, nanovesicles (gADNVs) were isolated from *Aloe* and used as functional carriers to encapsulate the photothermal agent indocyanine green (ICG). The constructed ICG/gADNVs drug delivery system exhibits excellent stability in both serum environment and long-term storage conditions, can effectively penetrate the skin barrier or target tumor tissue, and has a significant photothermal killing effect on melanoma under laser irradiation, and its tumor inhibition effect is better than that of free ICG and traditional ICG liposomes [87].

Further, the researchers used natural *Bitter melon*-derived extracellular vesicles (BMEVs) and 5-fluorouracil (5-FU) to construct a synergistic therapeutic nanoplatform through sonication. The platform not only induces S-phase cycle arrest and apoptosis in oral squamous cell carcinoma cells by inducing reactive oxygen species production and upregulation of JUN protein, but also downregulates the expression of NLRP3 inflammasomes with the help of RNA components it carries, thereby reversing the inflammatory resistance pathway activated by 5-FU, showing enhanced tumor growth inhibition and effectively reducing chemotherapy resistance in both in vitro and in vivo models [88].

In addition, a novel nanodrug-loading platform with good biocompatibility, high cell uptake efficiency, and natural targeting was constructed based on high-purity exosome-like nanovesicles derived from celery (*Apium graveolens* L.), which were successfully loaded with doxorubicin through simple incubation and centrifugation purification strategies, and demonstrated significant tumor proliferation inhibition in both in vitro and in vivo models [89].

In terms of nucleic acid drug delivery, a new biological delivery system has been constructed by efficiently loading siRNAs targeting the DDHD1 gene into *Tangerine*-derived nanovesicles (TNVs) through electroporation. The platform can effectively deliver therapeutic siRNA to human colorectal cancer cells SW480 and significantly reduce DDHD1 gene expression, thereby inhibiting tumor cell proliferation [90].

In the field of synergistic immunotherapy, a study separated natural nanovesicles (GC NV) from the root of wild *Glycyrrhiza uralensis fisch* roots, and then used electroporation technology to encapsulate the STING agonist DMXAA inside the vesicles, and at the same time covalently modify the PD-L1 antibody on the surface of the vesicles through the DSPE-PEG-NHS linker to construct a multifunctional synergistic nanoplatform (GP@DMX NV). The platform can not only achieve targeted delivery and block the PD-1/PD-L1 immunosuppressive pathway through surface antibodies, but also release DMXAA to activate STING signals in the cell to promote dendritic cell maturation, and directly induce apoptosis using GC NV’s own active ingredients (such as miR2916 and isoglycyrizizin), ultimately achieving immune microenvironment remodeling and tumor growth inhibition in melanoma models [68].

In addition, *tea* leaf-derived nanovesicles (TLNVs) are used to load iron-based components with their inherent iron chelating ability to build an “iron supply amplifier” platform. The system induces immunogenic death (ICD) in tumor cells through the iron-dependent oxidative stress pathway, releases tumor-associated antigens, and effectively reprograms tumor-associated macrophages (TAMs) from immunosuppressive M2 phenotypes to anti-tumor M1 phenotypes, thereby synergistically enhancing the infiltration and killing activity of cytotoxic T lymphocytes in head and neck squamous cell carcinoma tissues to achieve efficient immune combination therapy [91].

In a study, a biomimetic nanoplatform is introduced employing pYEEIE peptide-functionalized exosome-like nanovesicles derived from *Rhodiola rosea* (RELNs) for the targeted delivery of doxorubicin (DOX), which demonstrates enhanced tumor accumulation, potent melanoma suppression, and a significantly improved safety profile by mitigating the cardiotoxicity associated with conventional chemotherapy [92].

Stec A et al. demonstrate that doxorubicin (DOX) incorporation into *Citrus limon*-derived extracellular vesicles can be precisely monitored using capillary electrophoresis and nanoplasmonic sensing, revealing drug accumulation in the vesicle’s interfacial region and yielding a formulation that maintains cytotoxicity against HeLa cervical cancer cells while significantly reducing toxicity to human embryonic kidney cells (HEK293T), suggesting enhanced selectivity [93].

Feng’s team innovatively developed a microneedle system integrating *Garlic*-derived nanovesicles and garlic polysaccharides. This system enables near-infrared light-triggered on-demand drug release, which not only induces pyroptosis via cleavage of GSDME protein and amplifies mitochondrial damage from photodynamic therapy, but also synergistically reprograms tumor-associated macrophages from an immunosuppressive M2 phenotype to a pro-inflammatory M1 phenotype. Thereby, it establishes a self-amplifying cell death pathway and remodels the immunosuppressive tumor microenvironment, leading to significant suppression of melanoma growth [94].

These studies have fully proved that plant-derived nanovesicles can not only be used as excellent natural drug carriers, facilitating the efficient delivery of chemotherapy drugs, nucleic acid drugs and immunomodulators through a variety of sophisticated drug delivery strategies, but also play a synergistic anti-tumor role in a variety of tumor models through multiple mechanisms such as direct killing, immunomodulation and reversal of drug resistance based on their own bioactive components and targeting characteristics, showing great clinical application prospects. To more clearly summarize and present the construction strategies, drug loading methods, and synergistic mechanisms of the aforementioned multifunctional delivery platforms, we have systematically categorized and organized these studies in Table 3.

## 7. Clinical Trial on PDNVs

Although preclinical studies have highlighted the substantial potential of plant-derived nanovesicles (PDNVs) in anti-tumor therapy and drug delivery, clinical translation remains in its infancy. According to *ClinicalTrials.gov*, only a limited number of trials have been conducted (Table 3), involving plant sources such as *Ginger* (NCT04879810), *Grape* (NCT01668849), *Turmeric* (NCT01294072), and *Citrus limon* L. (NCT04698447), targeting conditions including inflammatory bowel disease, oral mucositis in head and neck cancer, colorectal cancer, and metabolic syndrome (Table 4).

From a translational perspective, currently registered PDNV-related clinical studies demonstrate a clear imbalance between therapeutic development and nutraceutical application. Among the reported trials, *Ginger* (NCT04879810) and *Grape* (NCT01668849) are conducted as disease-oriented interventional studies within a pharmacological treatment framework, whereas *Turmeric* (NCT01294072) and *Citrus limon* L. (NCT04698447) are primarily evaluated as dietary supplements for supportive care. These differing clinical objectives inherently correspond to distinct regulatory pathways and reference standards, including divergent GMP requirements and quality control criteria. Such inconsistency not only complicates regulatory alignment (e.g., FDA/EMA frameworks) but also poses challenges for pharmaceutical-grade standardization and large-scale industrial production.

In summary, although clinical research on plant-derived nanovesicles (PDNVs) remains limited, early evidence suggests potential value in therapeutic applications and drug delivery. However, existing clinical trials are generally small in sample size, lack systematic comparative analyses across diverse human subpopulations, and employ relatively restricted evaluation metrics, thereby limiting comprehensive assessment of their clinical relevance and benefit.

## 8. Prospects and Challenges

### 8.1. Comparative Safety and Translational Potential of PDNVs

Plant-derived nanovesicles (PDNVs) show promise as next-generation nanocarriers. Despite early-stage clinical translation, PDNVs present a unique profile balancing biosafety, functional versatility, and translational feasibility compared to synthetic and bioinspired nanomaterials. Despite well-defined biogenesis pathways and relatively predictable pharmacokinetics, mammalian exosomes may elicit immune responses under pathological or repeated exposure conditions, and the difficulty of ensuring fully healthy donors poses an additional challenge for their reliable collection [96]. In contrast, PDNVs isolated from edible plant sources such as *cloudberries* have demonstrated low immunogenicity and minimal immune perturbation in vivo, with negligible elevations in inflammatory markers and no histopathological toxicity following repeated dosing [97]. Synthetic nanocarriers, including PEGylated lipid nanoparticles and polymeric systems, permit precise control over particle size, composition, and drug loading but are frequently associated with innate immune recognition and complement activation, as correlations between pre-existing anti-nanoparticle immunoglobulins and variable complement responses in human plasma have demonstrated [98]. By comparison, edible plant-derived exosome-like nanovesicles administered in murine models delivered antigen effectively with minimal systemic toxicity, consistent with a favorable safety profile [99]. Hybrid biomimetic nanoparticles enhance targeting efficiency and circulation time but may exhibit unpredictable immunological interactions due to fabrication complexity and membrane-source variability; macrophage membrane-coated systems have been shown to activate macrophages and T cells in vivo, indicating a risk of unintended immune activation and inter-batch biological variability [100]. In contrast, edible plant-derived nanovesicles such as *cucumber*-derived PDNVs exhibited effective in vivo delivery with minimal systemic toxicity and no overt tissue damage, highlighting an inherently favorable safety profile [72].

Therefore, with their superior biological activity and notably low immunogenicity, plant-derived nanovesicles (PDNVs) demonstrate broader clinical application prospects compared to traditional nanomedicines. Their natural origin, minimal immune activation risk, and favorable engineering adaptability collectively form a solid foundation for their long-term development.

### 8.2. Key Challenges Limiting the Clinical Translation of Plant-Derived Nanovesicles

Notwithstanding their promising outlook, the clinical translation of plant-derived nanovesicles (PDNVs) faces interconnected challenges related to ethical considerations, administration routes, industrial standardization, and conceptual definition. Ethical concerns extend beyond source heterogeneity—arising from differences between artificially cultivated and wild-grown plants and from geographic variability affecting PDNV composition and therapeutic consistency—to fundamental departures from traditional herbal therapeutic principles. Conventional herbal medicine is typically based on multi-herb formulations established through long-term empirical practice, in which synergistic interactions and mutual detoxification help balance efficacy and toxicity. In contrast, PDNV-based interventions generally rely on nanovesicles derived from a single plant source, a strategy that may amplify therapeutic effects while simultaneously magnifying organ-specific or systemic toxicities. These concerns are further compounded by the predominant use of fresh plant materials in PDNV studies, exposing patients to intact proteins and nucleic acids—particularly non-coding RNAs—whose biological effects in humans remain insufficiently characterized. In addition, in vivo behavior remains poorly defined, as oral administration is influenced by inter-individual variability in digestion and gut microbiota, whereas intravenous delivery introduces uncertainties related to systemic biodistribution, immune recognition, clearance, and long-term safety. Finally, although multiple terms coexist in the literature—including PDNVs, plant-derived extracellular vesicles (PDEVs), extracellular vesicles (EVs), and nanovesicles (NVs)—most plant nanovesicles are obtained through mechanical disruption or culture-based methods and do not strictly meet the criteria for mammalian exosomes; therefore, the term “plant-derived nanovesicles (PDNVs)” is adopted here, and the absence of standardized manufacturing and characterization workflows further underscores the need for unified technical and regulatory frameworks to enable reliable clinical translation. Taken together, addressing these ethical, biological, technical, and conceptual challenges in a coordinated manner will be essential for the responsible, reproducible, and clinically meaningful development of PDNV-based therapeutics.

## 9. Conclusions

As a type of natural nanoscale vesicles derived from plants, physical nanovesicles have the unique advantages of high biocompatibility, strong intrinsic biological activity and easy large-scale production, showing great application potential in the fields of drug delivery and disease treatment. However, it should be noted that many of the studies cited in this review obtained plant nanovesicle preparations through enrichment-based isolation methods, and these preparations may be more appropriately regarded as crude or partially purified fractions rather than rigorously purified vesicles. Therefore, although their clinical transformation still faces challenges such as standardization problems caused by heterogeneous sources, immature large-scale production processes, insufficient in vivo targeting, and unclear regulatory paths, through the establishment of quality control systems, innovation in engineering modification strategies, and collaborative promotion by industry, academia and research, plant nanovesicles are expected to overcome existing bottlenecks, play an important role in the development of precision medicine and green therapies in the future, and ultimately achieve a successful leap from basic research to clinical application.

## Figures and Tables

**Table 1:** Isolation protocol for PDNVs.

Method	Advantages	Shortcoming	References
Ultracentrifugation	Considered the “gold standard”, it is easy to operate, does not require the introduction of exogenous reagents, and can handle large sample volumes.	Purity is limited, and ultracentrifugation may cause vesicle damage.	[46]
Density gradient centrifugation	The purity is high, which can effectively separate vesicles and impurities, and the buffering effect of the gradient medium can reduce vesicle damage.	The process is complex and time-consuming, and the introduced gradient medium may cause pollution and high cost.	[47,48]
Ultrafiltration centrifugation	Simple and fast, does not affect biological activity.	Blockage or contamination may damage vesicle membrane structures.	[54]
Dimensional exclusion chromatography	High purity and can maintain vesicle structure and biological activity well.	High cost, high technical requirements, low harvest.	[22,50]
Polymer precipitation method	Easy operation, high recovery rate, low equipment requirements.	Low purity, many impurities, and vesicles are easily contaminated by polymers.	[53]

**Table 2:** Summary of tumor inhibition mechanism of PDNVs.

Mode of Action	Plants	Target	Mechanism	References
Cell cycle/apoptosis pathway	*Asparagus cochinchinensis*	hepatocellular carcinoma	Induce apoptosis of tumor cells.	[74]
	*Citrus limon*	Chronic myeloid leukemia	Activation of TRAIL-mediated apoptosis.	[75]
	*Onion*	Prostate cancer, cervical cancer	Mitochondrial apoptosis, cycle arrest.	[76]
	*Garcinia mangostana* L.	Glioma	Apoptosis and value-added inhibition.	[29]
	*Centella asiatica*	hepatocellular carcinoma	ROS production, mitochondrial damage, and cycle block	[32]
	*Grapes*	Breast cancer	Cycle arrest and apoptosis	[30]
Immune regulation	*Ginseng*	Melanoma	M2 cells polarize to M1 cells.	[77]
	*Platycodon grandiflorum*	Breast cancer	Polarization of tumor-associated macrophages (TAMs) towards the M1 phenotype.	[78]
	*Garlic*	Multiple cancers	Enterogenic γδ T cell translocation.	[79]
	*Cannabis sativa* Roots	Lymphoma	Activate DC, NK cells, and Th1 cells, downregulate Treg cells, and promote CTL differentiation.	[80]
	*Artemisia annua*	Lung cancer	Activation of the cGAS-STING pathway promotes polarization of M2 cells to M1 cells.	[33]
Metabolic regulation	*Biyang floral mushroom*	Normal cells	Antioxidant, hematopoietic system protection, organ damage repair, DNA protection.	[57]
	*Honeysuckle*	kidneys	Reduces oxidative stress and protects mitochondrial function.	[82]
	*Momordica charantia* L.	Cardiomyocytes	Inhibition of ubiquitinated degradation of p62 protein to relieve Keap1 inhibition of Nrf2, thereby mitigating cellular oxidative stress, mitochondrial damage, and apoptosis.	[83]
	*Beta vulgaris*	Cardiomyocytes	Inhibition of ferroptosis.	[84]
	*Ginger*	Melanoma	Inhibition of PLC expression in bacteria, leading to (DHA) accumulation, and enhancing the therapeutic efficacy of PDL1 antibody.	[48]

**Table 3:** Overview of PDNVs in drug delivery and antitumor applications.

Plant	Therapeutic Agent	Modification Strategy	Function	Outcome	Reference
*Grapefruit*	Doxorubicin (DOX)	Surface modification with cRGD peptide + heparin nanoparticles	Crosses BBB/BTB, targeted tumor accumulation	Glioma models, significant tumor inhibition	[86]
*Aloe vera*	Indocyanine green (ICG)	Functional encapsulation	Photothermal tumor cell killing	Melanoma models, superior to free ICG and liposomes	[87]
*Bitter melon*	5-FU	Sonication loading	S-phase arrest, apoptosis; downregulates NLRP3, reverses chemotherapy resistance	Oral squamous carcinoma cells and in vivo models	[88]
*Apium graveolens*	Doxorubicin	Incubation + centrifugation purification	Natural targeting, high cellular uptake	In vivo models, significant tumor growth inhibition	[89]
*Tangerine*	siRNA (DDHD1)	Electroporation	Gene silencing inhibits tumor cell proliferation	Human colorectal cancer SW480 cells	[90]
*Glycyrrhiza uralensis*	DMXAA + PD-L1 antibody	Electroporation for drug loading; surface covalent antibody conjugation	Activates STING signaling, PD-1/PD-L1 blockade, induces apoptosis	Melanoma models, immune microenvironment remodeling	[68]
*Tea leaf*	Iron-based compounds	Intrinsic iron chelation	ICD induction; TAM M2→M1 polarization; enhances T cell cytotoxicity	Head and neck squamous cell carcinoma	[91]
*Rhodiola rosea*	Doxorubicin	pYEEIE peptide functionalization	Enhanced tumor accumulation, reduced cardiotoxicity	Melanoma models	[92]
*Citrus limon*	Doxorubicin	Fluorescence/electrophoresis monitoring	Precise drug localization, enhanced selectivity	HeLa & HEK293T cells	[93]
*Garlic*	Photosensitizer + polysaccharide	Microneedle system + NIR-triggered release	M2→M1 polarization	Melanoma models	[94]

**Table 4:** The clinical trials of PDNVs.

Plant	Intervention/Masking	Application	Aim	State	Number
*Ginger*	As Treatment, Open Label	Inflammatory Bowel Disease	Compare the symptoms of Inflammatory Bowel Disease (IBD)	Completed	NCT04879810
*Grape*	As Dietary Supplement, Open Label	Head and neck cancer oral mucositis	Immune responses to tumor antigens, metabolic and molecular markers	Completed	NCT01668849
*Turmeric*	As Dietary Supplement, Open Label	Colon Cancer	Effect of immune modulation, cellular metabolism, and phospholipid profile	Unknown	NCT01294072
*Citrus limon* (L.)	As Dietary Supplement, Double-blind study	Metabolic Syndrome	Improve different cardio-metabolic parameters in subjects with metabolic syndrome	Completed [95]	NCT04698447

## Data Availability

Not applicable.
